# A novel cryoprecipitate-enriched PRP (Cryo-PRP) gel with enhanced mechanical strength and regenerative capacity accelerates enterocutaneous fistula healing

**DOI:** 10.3389/fbioe.2025.1668608

**Published:** 2025-10-08

**Authors:** Zhang-Sheng Zhao, Zhen-Zhen Wang, Hua Ye, Xian-Lei Cai, Bin Hu, Qiu Han, You-Li Ma

**Affiliations:** ^1^ Department of Blood Transfusion, The Affiliated LiHuiLi Hospital of Ningbo University, Ningbo, Zhejiang, China; ^2^ Department of Infectious Diseases and Liver Diseases, The Affiliated LiHuiLi Hospital of Ningbo University, Ningbo, Zhejiang, China; ^3^ Department of Gastrointestinal Surgery, The Affiliated LiHuiLi Hospital of Ningbo University, Ningbo, Zhejiang, China; ^4^ Department of Clinical Laboratory, The Affiliated LiHuiLi Hospital of Ningbo University, Ningbo, Zhejiang, China; ^5^ Zhejiang International Joint Laboratory of Advanced Membrane Materials & Processes, Ningbo Institute of Materials Technology and Engineering, Chinese Academy of Sciences, Ningbo, Zhejiang, China

**Keywords:** cryoprecipitate, platelet-rich plasma, enterocutaneous fistula, tissue regeneration, angiogenesis

## Abstract

**Introduction:**

Enterocutaneous fistula (ECF) remains a major clinical challenge due to its complex pathophysiology, high recurrence, and limited non-surgical options. Platelet-rich plasma (PRP) has demonstrated regenerative potential, but its limited mechanical strength restricts its application in high‐output fistulas. To overcome this limitation, we developed a cryoprecipitate‐enriched PRP (Cryo‐PRP) with enhanced fibrinogen content and gel stability.

**Methods:**

Cryo‐PRP was prepared by cryoprecipitation of conventional PRP. Characterization included fibrinogen quantification, thromboelastography (TEG), and scanning electron microscopy (SEM). Therapeutic efficacy was assessed in a rat ECF model by evaluating fistula closure, histological changes, and expression of angiogenic and inflammatory markers.

**Results:**

Cryo‐PRP exhibited significantly higher fibrinogen levels (5.26 ± 0.78 g/L vs. 2.58 ± 0.49 g/L, P < 0.001) and greater clot firmness (TEG‐MA: 37.8 ± 2.2 mm vs. 28.7 ± 1.3 mm, P < 0.001) compared with standard PRP. SEM revealed a denser and more organized fibrin network in Cryo‐PRP gels. In vivo, Cryo‐PRP accelerated wound healing, enhanced epithelialization, preserved crypt architecture, and reduced inflammation. Immunostaining demonstrated increased neovascularization (CD34), upregulation of regenerative markers (α‐SMA, CD31, VEGF, PCNA), and suppression of TNF-α expression.

**Discussion:**

Cryo‐PRP demonstrates superior mechanical and biological properties over conventional PRP, effectively promoting tissue regeneration and reducing inflammation in an ECF model. These findings support Cryo‐PRP as a safe, autologous, and minimally invasive therapeutic strategy for ECF management.

## 1 Introduction

Enterocutaneous fistula (ECF) is an abnormal connection between the gastrointestinal (GI) tract and the skin, most frequently occurring as a postoperative complication (Gefen et al., 2022). ECF is associated with a high risk of infection, fluid and electrolyte imbalances, malnutrition, hemorrhage, and multiorgan dysfunction (Denicu et al., 2022). Its management poses significant clinical challenges due to its prolonged disease course, poor prognosis, high recurrence and mortality rates, and profound impact on patients’ quality of life (Chan et al., 2022). Although surgical resection and reanastomosis can be effective in selected cases, these procedures are often poorly tolerated, and anastomotic failure frequently leads to recurrent fistulas (Ghimire, 2022). Consequently, there is an urgent need for a safe, effective, and minimally invasive non-surgical treatment.

Fibrin glue (FG), derived from human or animal plasma, has been widely used as a hemostatic and tissue adhesive agent (Spotnitz, 2010). Its three-dimensional fibrin network provides a scaffold for endothelial and fibroblast cell migration and proliferation, thereby facilitating tissue repair (Li et al., 2024). In the 1980s, Groitl et al. (Groitl and Scheele, 1987) first applied FG endoscopically for GI fistula closure, pioneering a non-surgical approach. Later studies showed that while FG alone has a closure success rate of around 36.5%, the rate improves to 55.7% when combined with other endoscopic methods (Lippert et al., 2011). However, the use of commercially available FG is limited by its allogeneic or xenogeneic origin, carrying risks of viral transmission and immunogenicity (Khodakaram-Tafti et al., 2017). Moreover, its therapeutic efficacy is constrained by the absence of growth factors essential for tissue regeneration, contributing to relatively high recurrence rates-particularly within the first 3 months post-treatment (Loungnarath et al., 2004).

Platelet-rich plasma (PRP), an autologous platelet concentrate prepared by density gradient centrifugation (Harrison et al., 2023), has gained attention as a biocompatible and regenerative material. Upon activation, PRP releases growth factors including platelet-derived growth factor (PDGF), transforming growth factor-beta (TGF-β), vascular endothelial growth factor (VEGF), insulin-like growth factor (IGF), and epidermal growth factor (EGF), which promote mesenchymal stem cell (MSC) differentiation, fibroblast proliferation, and extracellular matrix (ECM) synthesis (Dos Santos et al., 2021). In addition, the intrinsic fibrinogen content of PRP allows it to form a 3D hydrogel structure upon activation by thrombin and calcium ions, making it an ideal scaffold for tissue repair (Napit et al., 2023). PRP also possesses antibacterial activity via platelet-derived antimicrobial peptides (Aquino-Domínguez et al., 2021).

Previous studies have demonstrated the successful application of platelet-rich plasma (PRP) gel in the treatment of alveolar fistulas (Andreetti et al., 2010), anal fistulas (de la Portilla et al., 2019), rectovaginal fistulas (Hermann et al., 2022), and vesicovaginal fistulas (Shirvan et al., 2013). In our hospital, we have also validated the therapeutic efficacy of PRP gel in simple enterocutaneous fistulas. However, its efficacy appears suboptimal in complex enterocutaneous fistulas characterized by persistent leakage of intestinal contents. We hypothesize that the primary cause of treatment failure may be the insufficient mechanical stability of PRP gel, making it susceptible to displacement by fistula exudate. To overcome this limitation, we propose enhancing the mechanical integrity of PRP gel by preparing a cryoprecipitate-enriched PRP (Cryo-PRP) using cryoprecipitation techniques (Sparrow et al., 2023). This approach may increase the concentration of fibrinogen in the gel, thereby improving its mechanical strength and providing a more stable matrix for the sustained release of growth factors, ultimately improving the healing outcomes of enterocutaneous fistulas.

To validate this hypothesis, we first quantified the fibrinogen content in Cryo-PRP and assessed its mechanical properties using thromboelastography (TEG). The ultrastructure of the gel was then examined using scanning electron microscopy (SEM). Finally, we established a rat model of enterocutaneous fistula to evaluate the *in vivo* therapeutic efficacy of Cryo-PRP gel.

## 2 Methods

### 2.1 Preparation of PRP and Cryo-PRP

A total of 10 healthy volunteers were recruited. For each volunteer, 30 mL of PRP was collected using a cell separator (Fresenius COM. TEC). The procedure was performed by physicians following standard operating protocols. Extracorporeal circulation was established through bilateral antecubital veins using 18-G intravenous catheters. Anticoagulation was maintained with ACD-A at a ratio of 1:7.5. The blood flow rate was approximately 40 mL/min, with a total circulating volume of 300–500 mL. 25 mL PRP was used for the preparation of Cryo-PRP. The procedure for the preparation of Cryo-PRP was as follows: PRP was first frozen at −80 °C for 24 h. Post-thawing at 4 °C with precipitate formation, centrifugation (3,000 rpm × 10 min) was conducted under isothermal conditions. After centrifugation, four-fifths of the supernatant (PRP-S) was carefully removed, and the remaining one-fifth was resuspended to obtain Cryo-PRP. PRP, Cryo-PRP, or PRP-S were mixed with a bovine thrombin/calcium gluconate solution at a volume ratio of 10:1. After 30 s of mixing, the corresponding gel form was obtained.

### 2.2 Scanning electron microscopy (SEM)

PRP, Cryo-PRP, and PRP-S gels were prepared as described and immediately fixed in 2.5% glutaraldehyde at 4 °C for 24 h. After fixation, the samples were washed three times with phosphate-buffered saline (PBS) and subsequently dehydrated through a graded ethanol series (30%, 50%, 70%, 90%, and 100%, 10 min each). The dehydrated samples were then dried using critical point drying, mounted on aluminum stubs, and sputter-coated with a thin layer of gold. Morphological observation was performed using a scanning electron microscope (SEM; Hitachi S-4800, Japan).

### 2.3 Thromboelastography (TEG) and fibrinogen (Fib) measurement

Thromboelastography (TEG) was performed to evaluate the coagulation properties of PRP, Cryo-PRP, and PRP-S using a TEG^@^5,000 Thromboelastography Hemostasis Analyzer (Haemonetics Corp., United States). For each sample, 340 μL of PRP, Cryo-PRP, or PRP-S was mixed with 20 μL of 0.2 M calcium chloride in a TEG cup and tested according to the manufacturer’s instructions. Parameters including clot formation time (K) and maximum amplitude (MA) were recorded and analyzed.

Plasma fibrinogen (Fib) levels were measured using an automated coagulation analyzer (Stago STA-R Evolution, Diagnostica Stago, France) based on the Clauss method. All measurements were performed in accordance with the manufacturer’s protocol.

### 2.4 Animal model and experimental design

Male Sprague-Dawley (SD) rats (6 weeks old, obtained from SiPeiFu Biotechnology Co., Ltd., license number SCXK 2024-0001) were housed under controlled environmental conditions: temperature 20–26 °C, relative humidity 40–70%, with a 12-h light/dark cycle.

After an acclimation period, an enterocutaneous fistula (ECF) model was established. Anesthesia was induced via intraperitoneal injection of xylazine hydrochloride (15 mg/kg) combined with Su-Mian-Xin (10 mg/kg). After routine skin preparation, a midline abdominal incision was made starting from the xiphoid process. Abdominal muscles were dissected, carefully avoiding vascular structures. Subcutaneous tissues were then bluntly separated from the incision midpoint toward the left abdominal region. A circular skin incision (0.5 cm in diameter) was created approximately 1 cm lateral to the midpoint on the left abdomen, also avoiding prominent vessels. The cecum was identified in the lower left abdomen, either superficially or deep within the cavity, and gently exteriorized through the midline incision using a sterile cotton swab. It was then passed subcutaneously through the previously created tract to protrude 0.5 cm through the left abdominal circular skin incision. The blind end of the cecum was transected, and the cecal stump was fixed to the circular skin incision using multiple interrupted sutures. Hemostasis was achieved with sterile gauze or cotton swabs. The surgical incision was closed in layers, and animals were placed on a heating pad in a supine position until recovery from anesthesia, after which they were returned to standard housing conditions. The intestinal fistula model was verified by injecting a contrast agent into the external opening of the fistula and performing X-ray barium contrast imaging.

After model confirmation, rats were randomly assigned to three groups:

Control group: fistula surface disinfected and irrigated with saline.
PRP group: fistula surface disinfected, followed by PRP gel application.
Cryo-PRP group: fistula surface disinfected, followed by Cryo-PRP gel application.

The injection was administered from the deepest portion of the fistula, with gradual withdrawal of the needle while injecting, until the entire tract was filled. The volume used was approximately 300 μL per application, administered once daily for a total of three consecutive days. Rats-derived PRP was prepared using the Landesberg method (Landesberg et al., 2000). Whole blood was first centrifuged at 200 × g for 10 min to collect the plasma supernatant, which was then centrifuged at 200 × g for 10 min to isolate the PRP layer. Cryo-PRP was prepared as described in Section 2.1. Healing progression was monitored, and rats were euthanized on day 7 for tissue harvesting. Animals were euthanized by intraperitoneal injection of an overdose of xylazine hydrochloride (30 mg/kg) combined with Su-Mian-Xin (25 mg/kg), ensuring deep anesthesia and humane death.

### 2.5 Hematoxylin and eosin (HE) staining

Fistula tissues were fixed and then dehydrated through a graded ethanol series, followed by clearing in xylene and paraffin embedding. Tissue sections were prepared using a microtome and baked on slides. After deparaffinization and rehydration, the sections were stained with hematoxylin for 3–5 min, rinsed in running tap water, differentiated with 1% hydrochloric acid in ethanol, and then blued in a bluing reagent. Subsequently, eosin staining was performed for 3–5 min. The sections were dehydrated, mounted, and observed under a light microscope (BX43, Olympus, Japan).

### 2.6 Immunofluorescence staining

Paraffin-embedded fistula tissue sections were baked, deparaffinized, and rehydrated, followed by antigen retrieval using citrate buffer. The sections were then permeabilized with 0.5% Triton X-100 at room temperature for 10 min and blocked with bovine serum albumin (BSA; Solarbio) at 37 °C for 30 min. Subsequently, the sections were incubated overnight at 4 °C with rabbit anti-CD34 antibody (BIOSS, bs-064R; 1:100 dilution). After washing, Cy3-conjugated secondary antibody (1:100) was applied and incubated at 37 °C for 45 min. Nuclei were counterstained with DAPI, and the tissue sections were sealed and observed under a fluorescence microscope (CKX53, Olympus).

### 2.7 Immunohistochemistry

Immunohistochemistry was performed to evaluate the expression of tumor necrosis factor-α (TNF-α), platelet endothelial cell adhesion molecule-1 (CD31), vascular endothelial growth factor (VEGF), α-smooth muscle actin (α-SMA), and proliferating cell nuclear antigen (PCNA) in the fistula tissue. Paraffin-embedded sections of rat fistula tissue were baked, deparaffinized, and rehydrated, followed by antigen retrieval using citrate buffer. After blocking with 5% bovine serum albumin (BSA), the tissue sections were incubated overnight at 4 °C with the following primary antibodies: rabbit anti-TNF-α (BIOSS, bs-0646R; 1:100), rabbit anti-CD31 (Servicebio, GB11063-2; 1:200), rabbit anti-VEGF (BIOSS, bs-1313R; 1:200), rabbit anti-α-SMA (BIOSS, bs-10196R; 1:200), and rabbit anti-PCNA (Proteintech, 10205-2-AP; 1:200). After washing, tissue sections were incubated with horseradish peroxidase (HRP)-conjugated goat anti-rabbit IgG (H + L) secondary antibody (ZSGB-BIO, ZB-2301; 1:100), followed by DAB chromogenic development. Hematoxylin was used for nuclear counterstaining. The tissue sections were then dehydrated, cleared, mounted, and observed under a light microscope (BX43, Olympus).

### 2.8 Statistical analysis

Statistical analyses and graphical representations were performed using GraphPad Prism version 8.0. Quantitative data are presented as mean ± standard deviation (X ± SD). One-way analysis of variance (ANOVA) was used to compare quantitative values among multiple groups. Differences were considered statistically significant when *P* < 0.05.

## 3 Results

**FIGURE 1:**
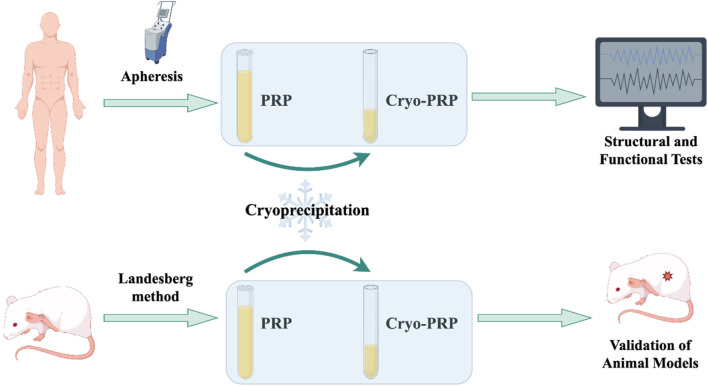
Technical roadmap of the study. This schematic flowchart illustrates the overall workflow of the study. Human volunteers were recruited for PRP collection, which was processed into PRP and Cryo-PRP. Human-derived PRP/Cryo-PRP was mainly used for *in vitro* analyses, including electron microscopy, thromboelastography, and fibrin structure characterization. Rat-derived PRP/Cryo-PRP was prepared following the Landesberg method and applied in animal experiments for *in vivo* validation.

**FIGURE 2:**
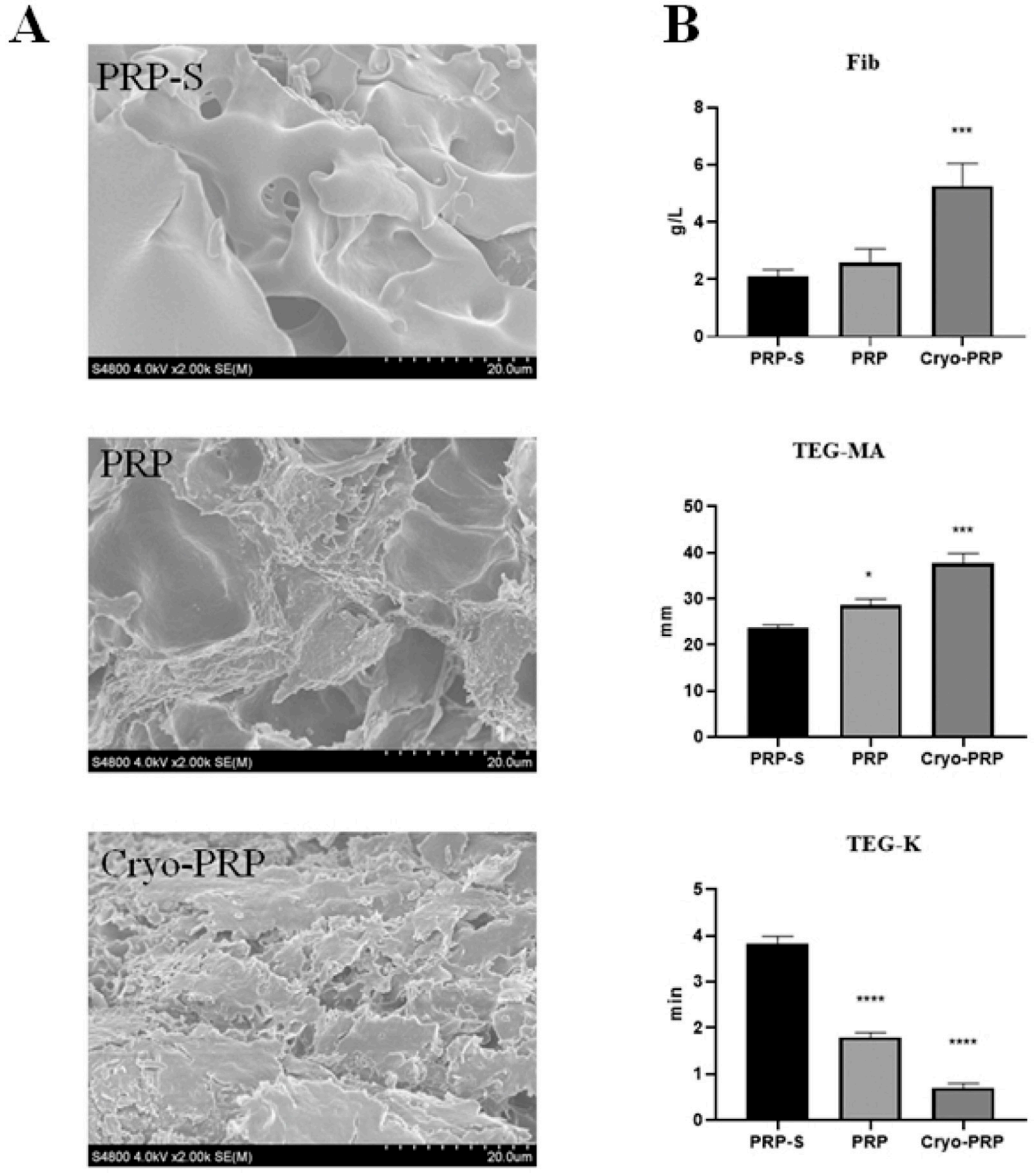
Structural and functional comparison of PRP-S, PRP, and Cryo-PRP. **(A)** Scanning electron microscopy (SEM) images of PRP-S, PRP, and Cryo-PRP showing distinct fibrin architectures. PRP-S exhibits a smooth surface with minimal fibrin network. PRP displays a loosely organized fibrin mesh. Cryo-PRP demonstrates a dense and compact fibrin matrix, indicating enhanced structural integrity. Scale bar = 20 μm. **(B)** Quantitative analysis of fibrinogen concentration (Fib), thromboelastography maximum amplitude (TEG-MA), and clot formation time (TEG-K) among the three groups. Cryo-PRP showed significantly higher fibrinogen levels and MA values, along with markedly shortened K time, compared to PRP-S and PRP. Data are presented as mean ± SD. Statistical significance: *P* < 0.05, ***P < 0.001, ****P < 0.0001.

The overall workflow of the study was schematically shown in Figure 1. The platelet count of PRP obtained by apheresis was 968 ± 204 × 10^9^/L. From 25 ml of PRP, an average of 5 ml (yield ∼20%) of Cryo-PRP was obtained through cryoprecipitation. Visually, Cryo-PRP exhibited increased turbidity compared to PRP and PRP-S, suggestive of denser protein content. Furthermore, we examined the microstructure of the three gels using SEM. The ultrastructural characteristics of PRP-S, PRP, and Cryo-PRP gels are shown in Figure 2A. PRP-S displayed a relatively smooth and loose fibrin network, while PRP exhibited a denser fibrin architecture with increased fiber interconnectivity. Cryo-PRP demonstrated a markedly compact and intricate fibrin meshwork, suggesting enhanced structural integrity and clot organization.

Quantitative analyses of fibrinogen concentration (Fib, g/L), thromboelastography maximum amplitude (TEG-MA, mm), and clot kinetics (TEG-K, min) for the three formulations are presented in Figure 2B. Cryo-PRP (5.26 ± 0.78 g/L) showed significantly higher fibrinogen levels compared to PRP (2.58 ± 0.49 g/L) and PRP-S (2.10 ± 0.24 g/L) (*P* < 0.001). TEG-MA was significantly higher in Cryo-PRP (37.8 ± 2.2 mm) and PRP (28.7 ± 1.3 mm) compared to PRP-S (23.7 ± 0.6 mm) (*P* < 0.001; *P* < 0.05), indicating increased clot strength. TEG-K, reflecting clot formation time, was significantly reduced in Cryo-PRP (0.7 ± 0.1 min) and PRP (1.8 ± 0.1 min) versus PRP-S (3.8 ± 0.2 min) (*P* < 0.0001), indicating more rapid clot formation.

**FIGURE 3:**
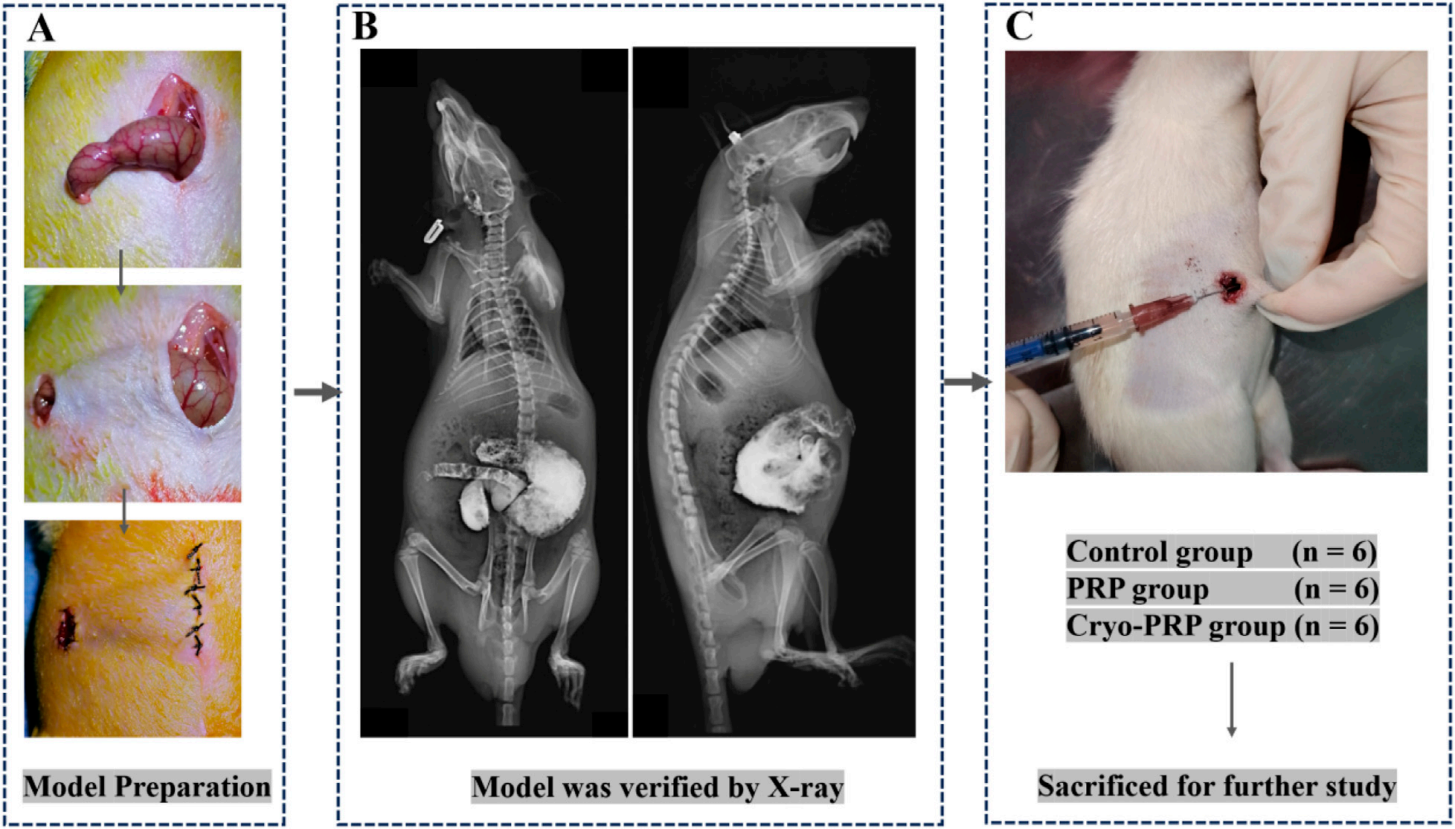
Schematic illustration of the animal experimental procedure. **(A)** The rat model of enterocutaneous fistula was established by making an incision at the end of the cecum and crossing the skin. **(B)** X-ray imaging was performed to verify the wound location and assess overall anatomical integrity. **(C)** Animals were randomly assigned to three groups: Control group (n = 6), PRP group (n = 6), and Cryo-PRP group (n = 6). After treatment administration, animals were sacrificed for subsequent histological and molecular analyses.

**FIGURE 4:**
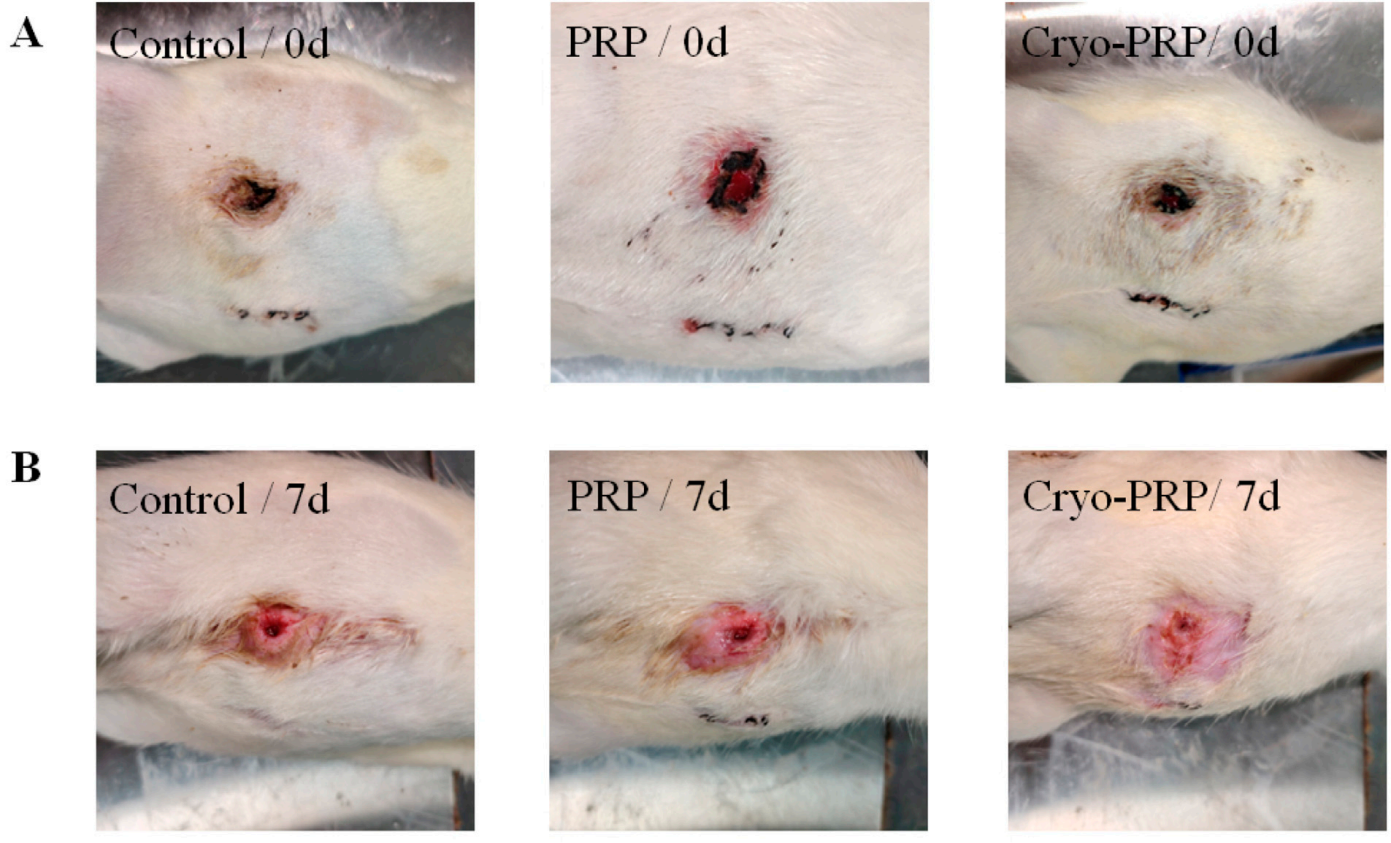
Representative wound images in each group at day 0 and day 7. **(A)** Photographs of enterocutaneous fistula at day 0 (immediately post-surgery) in the Control, PRP, and Cryo-PRP groups. **(B)** Corresponding wound sites at day 7 post-treatment. Compared to the Control group, wounds treated with PRP and Cryo-PRP exhibited accelerated healing with reduced wound size and enhanced epithelialization. Notably, the Cryo-PRP group demonstrated superior wound closure compared to the PRP group.

Subsequently, we established a rat model of enterocutaneous fistula to evaluate the therapeutic effect of Cryo-PRP. A schematic representation of the model is shown in Figure 3. The therapeutic effects of PRP and Cryo-PRP on fistula healing are presented in Figure 4. On day 0, all wounds exhibited open fistulas with no significant differences in initial appearance, accompanied by the exudation of intestinal contents. By day 7, the Cryo-PRP group showed markedly enhanced healing, characterized by reduced wound dimensions and decreased exudate, compared to both the PRP and control groups.

**FIGURE 5:**
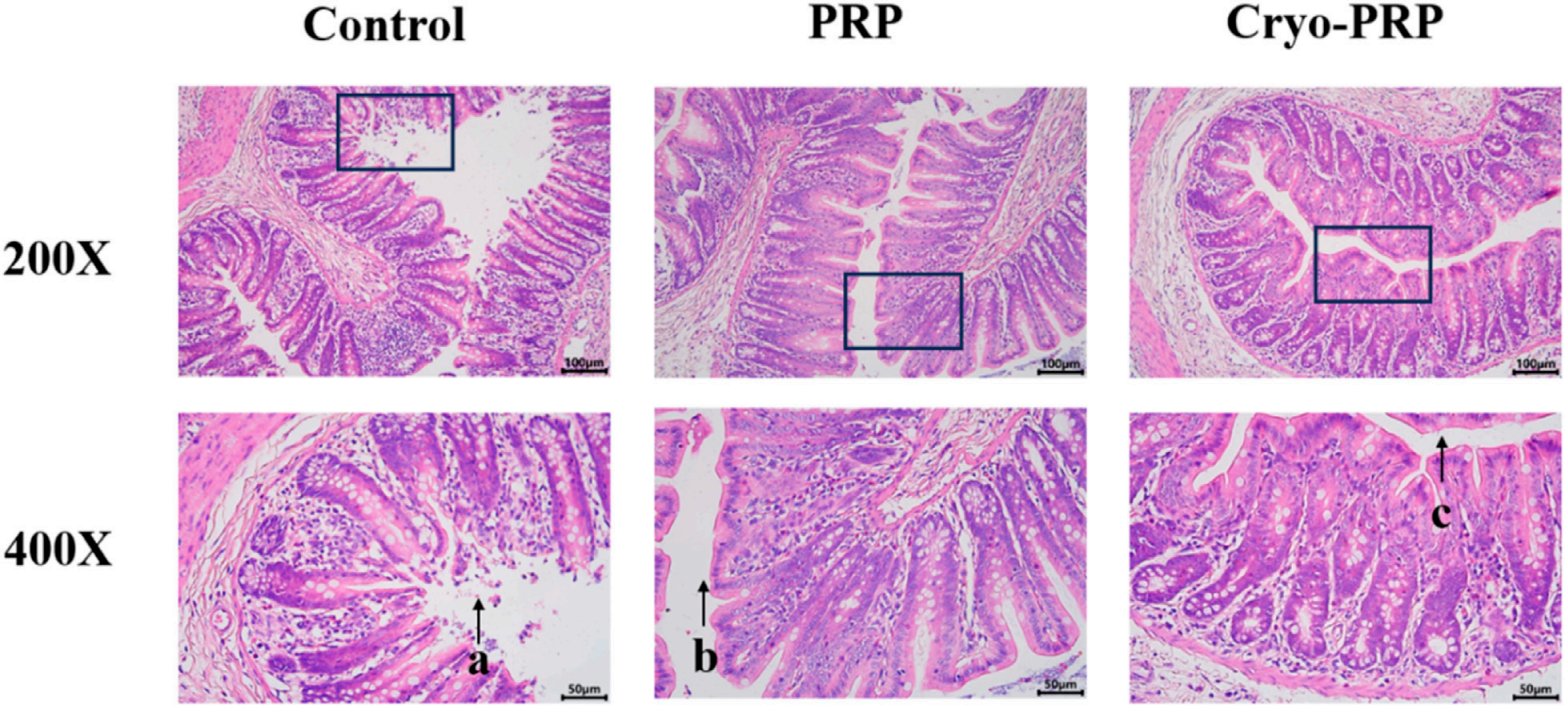
Histological evaluation of tissues in a fistula model by hematoxylin and eosin (H&E) staining. Representative histological images of fistula tissues from the Control, PRP, and Cryo-PRP groups at 200× (upper panel) and 400× (lower panel) magnification. In the Control group, extensive mucosal damage, disorganized crypt architecture, and dense inflammatory cell infiltration are evident (arrow a). In the PRP group, partial restoration of crypt structure and a moderate reduction in inflammatory infiltration are observed (arrow b). In the Cryo-PRP group, mucosal architecture appears largely intact, with well-organized crypts and minimal inflammatory cell presence (arrow c). Scale bars: 100 μm (200×), 50 μm (400×).

Hematoxylin and eosin (H&E) staining was performed to assess histopathological changes in fistula tissues among the Control, PRP, and Cryo-PRP groups. In the control group, severe mucosal damage was observed, including disorganized crypt architecture, epithelial erosion, and pronounced inflammatory cell infiltration within the lamina propria and submucosa. These findings indicate ongoing inflammation and compromised tissue integrity. In contrast, PRP-treated tissues showed partial restoration of mucosal architecture, with more organized crypts and reduced inflammatory infiltration, suggesting moderate attenuation of inflammation and early tissue repair. Notably, Cryo-PRP-treated tissues exhibited near-complete preservation of crypt structure, regularly arranged glands, and an intact epithelial layer. Inflammatory cell presence was minimal, and submucosal edema was significantly reduced, indicating effective resolution of inflammation and advanced tissue regeneration (Figure 5).

**FIGURE 6:**
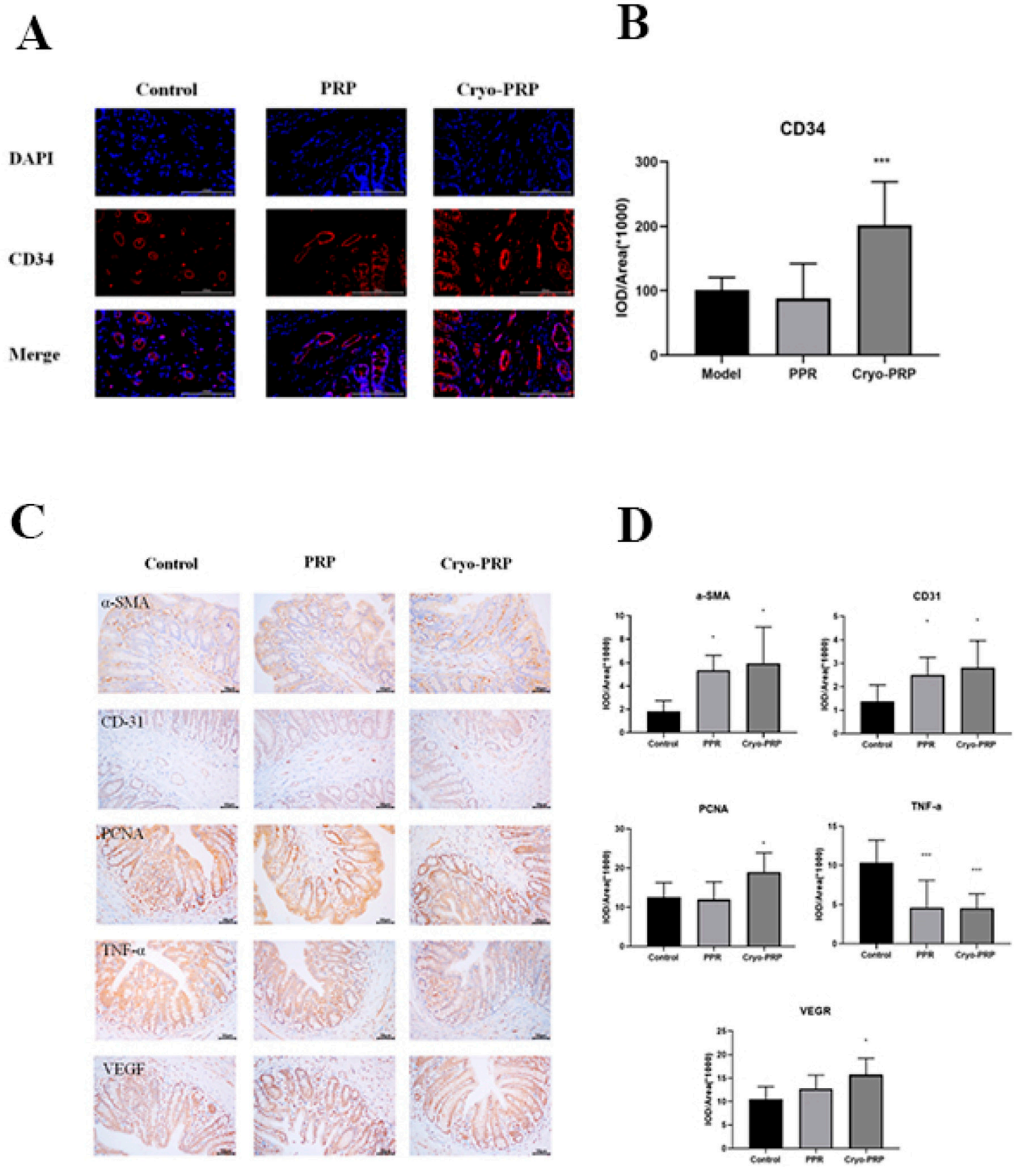
Immunofluorescence and immunohistochemical analysis in fistula tissues. **(A)** Representative immunofluorescence staining of CD34 (red) and DAPI (blue) in fistula tissues from Control, PRP, and Cryo-PRP groups. **(B)** Quantitative analysis of CD34 expression using integrated optical density (IOD/Area). **(C)** Representative images of immunohistochemical staining for α-SMA, CD31, VEGF, PCNA, and TNF-α in the Control, PRP, and Cryo-PRP groups are shown (original magnification: ×400). Expression of α-SMA, CD31, VEGF, and PCNA was markedly increased in the PRP and Cryo-PRP groups compared to Control, with the highest levels observed in the Cryo-PRP group. In contrast, TNF-α expression was significantly reduced following PRP and Cryo-PRP treatment, with minimal staining in the Cryo-PRP group. **(D)** Quantitative analysis of immunohistochemical staining using integrated optical density per area (IOD/Area × 10^6^). Data are presented as mean ± SD. *P < 0.05 vs. Control; ***P < 0.001 vs. Control.

CD34, a well-established marker of endothelial progenitor cells and neovascularization, was used to evaluate angiogenic activity in the fistula microenvironment. Immunofluorescence analysis revealed significantly higher CD34 expression in the Cryo-PRP group compared to the PRP and control groups (Figures 6A, B). Collectively, these findings indicate that Cryo-PRP augments neovascularization via enhanced progenitor cell recruitment.

To explore the mechanisms underlying the therapeutic effects of PRP and Cryo-PRP, immunohistochemical staining was performed for markers of myofibroblasts (α-SMA), angiogenesis (CD31 and VEGF), cell proliferation (PCNA), and inflammation (TNF-α). α-SMA, indicative of activated myofibroblasts involved in wound contraction and matrix remodeling, was significantly upregulated in both PRP and Cryo-PRP groups, with the highest expression observed in Cryo-PRP-treated tissues, suggesting robust tissue remodeling activity. CD31 and VEGF, markers associated with endothelial cells and angiogenic signaling respectively, were markedly elevated in the PRP group and further enhanced in the Cryo-PRP group, indicating increased neovascularization and improved tissue perfusion. Similarly, PCNA staining revealed strong nuclear positivity in both treatment groups, with Cryo-PRP eliciting the most pronounced proliferative response, reflecting accelerated cellular regeneration. In contrast, TNF-α, a key pro-inflammatory cytokine linked to chronic inflammation and tissue injury, was significantly downregulated in PRP and Cryo-PRP groups relative to the control, with Cryo-PRP demonstrating the most substantial reduction (Figure 6C). Quantitative image analysis further validated these findings, showing marked increases in the IOD/area values of α-SMA, CD31, VEGF, and PCNA (*P* < 0.05), and a significant decrease in TNF-α levels, particularly in the Cryo-PRP group (*P* < 0.001) (Figure 6D). Collectively, these results suggest that Cryo-PRP exerts superior regenerative effects by concurrently enhancing fibroblast activation, promoting angiogenesis, stimulating epithelial and stromal cell proliferation, and suppressing inflammatory responses — coordinated biological processes essential for efficient fistula healing. These findings underscore the potential of Cryo-PRP as a more potent and comprehensive therapeutic strategy compared to standard PRP.

## 4 Discussion

Platelet-rich plasma (PRP), an autologous blood-derived product known for its ability to promote tissue repair, has attracted considerable attention in the field of biomaterials. Its clinical indications in surgery have expanded progressively, from the treatment of chronic wounds to more complex scenarios such as fistula repair. However, conventional PRP contains only physiological levels of fibrinogen, and its gel form is characterized by a high water content and limited mechanical strength (Lian et al., 2024). In certain clinical situations, the PRP gel may be prematurely washed away by bodily fluids before exerting its intended biological effects, thereby limiting the time window for biological activity and compromising its regenerative potential. To address this limitation, cryoprecipitate-enriched PRP (Cryo-PRP), which contains a higher concentration of fibrin and retains a substantial amount of growth factors, has been proposed. This modification not only enhances the mechanical stability of the gel matrix but may also amplify its regenerative efficacy. However, current literature on Cryo-PRP remains extremely limited, with only a single clinical case report (Ren et al., 2012) and a hypothesis-driven review (Burnouf et al., 2013), and no prior studies have provided *in vivo* validation of its therapeutic potential.

To address this knowledge gap, we developed a Cryo-PRP gel enriched with fibrinogen and improved mechanical integrity, and for the first time, evaluated its therapeutic efficacy in an *in vivo* rat model of enterocutaneous fistula (ECF). Structurally, Cryo-PRP displayed a visibly denser and more turbid appearance than standard PRP and PRP-S, which was corroborated by SEM. The fibrin meshwork in Cryo-PRP was notably more compact and interconnected, indicating enhanced gel integrity and resistance to dissolution. This structural advantage was further supported by quantitative analyses: Cryo-PRP showed significantly elevated fibrinogen levels and superior thromboelastography (TEG) parameters, including increased maximum amplitude (TEG-MA, *P* < 0.001, Figure 2B) and reduced clot formation time (TEG-K, *P* < 0.0001, Figure 2B). These findings confirm that cryoprecipitate enrichment markedly enhances the mechanical elasticity of PRP gels, consistent with the increased mechanical strength observed in PRP-derived fibrin matrices (FM) prepared through ethanol-precipitation-based fibrinogen enrichment (Delgado et al., 2024). However, the cryoprecipitation method may better preserve bioactive molecules present in blood.

To evaluate the biological performance of Cryo-PRP, we established a rat model of enterocutaneous fistula. By day 7, rat ECF models treated with Cryo-PRP demonstrated accelerated wound contraction, decreased inflammation, and more complete epithelial coverage. Histopathological evaluation further revealed near-normal crypt architecture, minimal inflammatory infiltration, and reduced edema in the Cryo-PRP group, highlighting its efficacy in repairing ECF. At the molecular level, Cryo-PRP induced robust angiogenic and regenerative responses. CD34 immunofluorescence staining showed markedly higher expression in the Cryo-PRP group (*P* < 0.001, Figure 6B), suggesting enhanced recruitment of endothelial progenitor cells and stimulation of neovascularization. Immunohistochemical analysis revealed upregulated expression of α-SMA, CD31, VEGF, and PCNA (*P* < 0.05, Figure 6D)-key markers of myofibroblast activation, endothelial proliferation, angiogenesis, and cell proliferation—particularly in the Cryo-PRP group. Conversely, TNF-α levels (*P* < 0.05, Figure 6D), a hallmark of chronic inflammation, were significantly suppressed in Cryo-PRP–treated tissues, indicating effective modulation of the local inflammatory milieu, suggesting that Cryo-PRP may not only facilitate tissue regeneration but also exert immunomodulatory effects that contribute to a pro-healing environment. This phenomenon was also comparable to the effect observed when cryoprecipitate was added to PRP, which enhances angiogenic capacity and thereby promotes the healing of chronic wounds (Etulain et al., 2018).

Collectively, our results indicate that Cryo-PRP serves not only as a mechanically resilient scaffold for tissue repair but also as a biologically active matrix that orchestrates key processes essential for fistula healing, including inflammation resolution, angiogenesis, fibroblast activation, and epithelial proliferation. The superiority of Cryo-PRP over conventional PRP may stem from its higher fibrinogen concentration, improved growth factor retention, and enhanced clot stability, which together establish a more favorable microenvironment for tissue regeneration. These findings align with previous reports on the regenerative potential of PRP-based therapies in fistulas, while also addressing the long-standing limitation of inadequate mechanical stability in conventional PRP. By reinforcing the structural integrity of PRP through cryoprecipitation, our strategy offers a feasible and clinically meaningful advancement in the non-surgical treatment of enterocutaneous fistulas, achieved without reliance on exogenous cells or synthetic scaffolds.

Nevertheless, this study has several limitations. Firstly, while the rat model provides valuable insight into *in vivo* efficacy, differences between rodent and human wound healing necessitate cautious interpretation when translating these findings to clinical settings. Secondly, the relatively small number of animals used may limit the statistical power of the results. Thirdly, long-term outcomes, including fistula recurrence, scarring, and intestinal functional recovery, were not assessed and require further investigation. Fourthly, although we did not directly quantify the growth factor content in Cryo-PRP, Rock et al. reported that the concentration of PDGF increased by 100-fold when cryoprecipitate-derived fibrinogen was combined with platelets (Rock et al., 2006), suggesting that Cryo-PRP is theoretically rich in growth factors. Finally, given that this work represents an early-stage experimental study, further large-scale and mechanistic investigations are warranted before clinical translation.

In conclusion, Cryo-PRP gel demonstrates significant advantages over conventional PRP by concurrently enhancing the mechanical integrity and biological activity essential for effective ECF healing. This study provides strong preclinical evidence supporting the potential of Cryo-PRP as a novel, autologous, minimally invasive therapy for fistula management. Future directions include standardizing Cryo-PRP preparation methods, elucidating downstream signaling pathways, and conducting randomized clinical trials to validate efficacy in humans.

## Data Availability

The original contributions presented in the study are included in the article/supplementary material, further inquiries can be directed to the corresponding author.
